# Impact of smoking on the quantity and quality of antibodies induced by human papillomavirus type 16 and 18 AS04-adjuvanted virus-like-particle vaccine – a pilot study

**DOI:** 10.1186/1756-0500-7-445

**Published:** 2014-07-11

**Authors:** Proscovia B Namujju, Emma Pajunen, Aline Simen-Kapeu, Lea Hedman, Marko Merikukka, Helja-Marja Surcel, Reinhard Kirnbauer, Dan Apter, Jorma Paavonen, Klaus Hedman, Matti Lehtinen

**Affiliations:** 1 School of Health Sciences, University of Tampere, Tampere, Finland; 2 National Institute for Health & Welfare, P.O. Box 310, 90101 Oulu, Finland; 3 Haartman Institute, University of Helsinki, Helsinki, Finland; 4 Helsinki University Central Hospital Laboratory Division, Helsinki, Finland; 5 Department of Dermatology (DIAID), Medical University Vienna, Vienna, Austria; 6 Family Federation Finland, Helsinki, Finland; 7 University of Helsinki, Helsinki, Finland

**Keywords:** Antibody, Avidity, Human papillomavirus, AS04 adjuvanted vaccines, Cotinine, PATRICIA, Finland

## Abstract

**Background:**

The AS04-adjuvanted bivalent L1 virus-like-particle (VLP) vaccine (Cervarix™) against infection with human papillomavirus (HPV) types 16/18 holds great promise to prevent HPV16/18 infections and associated neoplasias, but it is important to rule out significant co-factors of the neoplasias like smoking.

**Methods:**

We conducted a pilot study to compare the quantity and quality of HPV16/18 antibody response at baseline and 7 months post vaccination in 104 non-smoking and 112 smoking female participants vaccinated at 0, 1 and 6 months with Cervarix™ (55 and 48 study participants) or with Hepatitis A vaccine (HAVRIX™) (48 and 64 participants, respectively). These 216 women were a sub-sample of 4808 baseline 16- to 17-year old Finnish women initially enrolled in the double-blind, randomized controlled phase III PATRICIA trial. Following end-of-study unblinding in 2009 they were randomly chosen out of all the participants of the three major Finnish PATRICIA study sites in the Helsinki metropolitan area (University of Helsinki, N = 535, and Family Federation Finland, N = 432) and Tampere (University of Tampere, N = 428). Following enrolment, serum samples were collected at month 0 and month 7 post 1st vaccination shot, and were analysed for levels and avidity of IgG antibodies to HPV16 and HPV18 using standard and modified (4 M urea elution) VLP ELISAs.

**Results:**

We found that at month 7 post vaccination women who smoked (cotinine level > 20 ng/ml) had levels of anti-HPV16/18 antibodies comparable to those of non-smoking women. Low-avidity HPV16/18 IgG antibodies were observed in 16% of the vaccinated women, and active smoking conferred a three-fold increased risk (95% CI 1.0-9.3) of having the low-avidity antibodies.

**Conclusion:**

Our data suggest that while smoking does not interfere with the quantity of vaccine-induced peak IgG levels, it may affect the avidity of IgG induced by HPV16/18 vaccination.

## Background

Infection with high-risk (hr) types of human papillomavirus (HPV) is the major cause of cervical cancer (CC) [1]. The necessary role of hrHPV infections in CC and other HPV related cancers provides an opportunity to significantly reduce associated disease burden by prophylactic vaccines [2] with proper coverage/herd immunity [3,4].

Important determinants of vaccine efficacy are the quantity and quality of the B-cell response. The AS04-adjuvanted HPV16/18 L1 virus-like-particle (VLP) vaccine induces high titer antibodies in adolescent and young women and men [5,6], able to neutralize the virus [7,8], and detectable up to 8.4 years post vaccination [9]. The immune responses are, however, not homogenous, eg. a proportion of HPV-16/18 vaccinated women, those with significantly lower serum antibody levels, had no detectable cervical antibodies 4 years post vaccination [6]. Furthermore, not all vaccinees develop high avidity antibodies, and the avidities and levels of the neutralizing antibodies correlate only moderately [10]. High avidity of HPV vaccine induced antibodies may indicate successful priming for long-term memory responses as previously suggested by Scherpenisse [11].

Smoking women have an impaired humoral immune response to HPV16/18 infections [12]. Smoking has also been associated with decreased clearance of persistent HPV lesions [13]. Furthermore, epidemiological studies have indicated that tobacco smoking is an independent risk factor for CC [14]. The effect of smoking on vaccine efficacy and effectiveness has been studied in influenza vaccine trials [15], but its influence on the HPV vaccine response is unknown. In this pilot study, we compared the quantity and quality of HPV16/18 antibody responses at baseline and seven months post vaccination in smokers and non-smokers vaccinated with three doses of AS04-adjuvanted HPV16/18 VLP vaccine or Hepatitis A vaccine.

## Methods

### Study participants

Enrolment for the PApilloma TRIal against Cancer In young Adults (PATRICIA (study trial number −580299/008)) study took place from April 2004 to May 2005 in Finland [16]. Healthy women aged 16–17 years were eligible to participate in the Finnish arm of this study with no exclusion criteria with regard to lifetime number of sexual partners before study enrolment [16,17]. Individuals with intact cervix, and agreeing to adequate contraception (barrier methods in combination with a spermicide, or hormonal contraception) over the vaccination period were eligible for inclusion. Exclusion criteria were limited to a history of colposcopy, pregnancy or breastfeeding, as well as autoimmune diseases and immunodeficiency. Informed consent was obtained from each participant at study baseline including later linkage to the Finnish Maternity Cohort (FMC) for the identification of serial serum samples post vaccination. The study protocols, recruitment material and informed consent forms were approved by the Finnish National and Pohjoispohjanmaan Sairaanhoitopiirin ethical review committees, and the retrieval of serum samples from the FMC repository by the National Institute for Health & Welfare.

### Study design

The PATRICIA study was a phase III double-blind, randomized controlled trial. In Finland, a total of 4,808 participants, were randomized in a 1:1 fashion with an internet-based centralized randomisation system, received either the AS04-adjuvanted HPV16/18 vaccine ((GlaxoSmithKline Biologicals, Rixensart, Belgium), (Each dose of HPV-16/18 L1 VLP AS04-adjuvanted candidate vaccine (Cervarix™) contained 20 mg each of HPV16 and HPV18 L1 proteins self-assembled as VLPs and adjuvanted with AS04 (50 μg 3-O-desacyl-40-monophosphoryl lipid A [MPL] and 500 μg aluminium hydroxide)) or, a control hepatitis A vaccine ((GSK Biologicals), Each dose of the control hepatitis A vaccine contained 720 ELISA units (EU) of inactivated hepatitis A antigen and 0 · 5 mg aluminium hydroxide)) to provide a health benefit for all participants and ensure double-blinding. Allocation of treatment numbers was stratified by study site and age.

A random sample of 216 study participants with unblinded individual vaccine allocation were selected for this study from the three largest Finnish study sites: Finnish Family Federation, Helsinki (432 participants, PI DA), University of Helsinki, Helsinki (535 participants. PI JP) and University of Tampere, Tampere (428 participants, PI ML). Serial serum samples accounting for the FMC-based long-term follow-up were available for 51, 97 and 103 participants, respectively, altogether for 251 of the total 1395 participants. The vaccines were supplied double-blinded in identical 0 · 5 ml pre-filled syringes and administered into the deltoid muscle on a 0, 1, and 6-month schedule.

### Serologic evaluation

Blood samples from the study participants were collected at baseline and at months 7 post vaccination, and an aliquot of each sample was stored at the National Institute for Health and Welfare laboratory in Oulu, Finland. Baseline and month 7 post vaccination trial serum samples were evaluated for HPV16 and HPV18 antibodies using a type-specific enzyme-linked immunosorbent assay (ELISA) as described [18-20]. Dilutions 1/30, 1/300 and 1/3000 were used to identify the linear part of the absorbance reactions for expression of the results as OD values.

### Avidity measurement

The avidity of IgG antibodies to HPV16 and HPV18 was evaluated in the HPV vaccine group (n = 103) using modified VLP ELISAs [21] Namujju et al. unpublished, (The VLPs were kindly donated by Dr Francis Dessy, GSK biologicals, Rixensart, Belgium). First, we screened the HPV16/18 vaccine induced antibodies for low avidity by single-dilution methods, using in parallel 4 M urea or 1 M ammonium thiocyanate as chaotropic agents. The month 7 post vaccination samples were diluted 1:1000 in assay diluent (PBS + 10% fetal bovine serum). Each sample was added in duplicate to a plate, one well in the plate was washed with PBS + 0.05% Tween 20 (PBST), and another well in the plate with either of the chaotropic agents. Blank well (assay diluent), negative and positive control pools for HPV6/11/16/18/31/33/45 natural infection derived antibodies, and acute-immunity (obtained from seroconverters within 6 months of natural infection) pools for HPV16 or HPV18 antibodies diluted 1:30 were included in each plate. After 2-hour incubation at room temperature (RT) the plates were washed three times with PBST and then (200 ul of) PBST was added in half of the plate, and (200 ul of) 4 M urea or 1 M ammonium thiocyanate in the other half for 15 minutes. The plates were again washed two times with PBST, after which the wells treated with the chaotropic reagents were rinsed extra two times with PBST. Thereafter the standard VLP ELISA procedures was followed [21]. The avidity index (AI) was calculated using the formula optical density (OD) of the chaotrope-treated well/OD of the PBST-treated well) × 100. The cut-off for low avidity (40%) was the mean + 3 standard deviations of acute- immunity controls.

Samples with results of low avidity in the single-dilution screening test were further analyzed by a serum titration approach [21-23], Namujju et al. unpublished, albeit using 4 M urea rather than 6 M urea. The samples were serially diluted at four fold steps; A) 1:100, B) 1:400, C) 1:1600, D) 1:6400 and E) 1:25600. After 2-hour incubation at RT, one half of the plate was washed with 4 M urea, and another half with PBST. Thus, from each sample two IgG-end-point titres at a cut-off of 0.200 (urea +/ urea -) were obtained. Their ration – representing IgG avidity – was calculated with curve-fitting software [24].

### Cotinine measurements

Serum samples were measured for cotinine using an immunoassay method (OraSure Technologies, Bethlehem, PA, USA) carried out as a quantitative assay based on the competition between free cotinine in the sample and horseradish peroxidase-labeled cotinine. Cotinine was quantified spectrophotometrically at 450 nm and 630 nm relative to a standard curve. The assay’s sensitivity is 95%–97% and specificity 99%–100% [25,26]. A cotinine level of 20 ng/ml was used as an indicator of an active smoker, according to the manufacturer.

### Statistical analyses

The pilot immunoanalyses included women who met eligibility criteria, received all vaccination shots and complied with the protocol procedures. Seropositivity rates of HPV16 and HPV18 were calculated. The Mann–Whitney test was used to compare the mean absorbances of anti-HPV16 and HPV18 antibodies between non-smokers and smokers in vaccine and control groups. For each antigen the mean absorbance level and standard deviation (±SD) were reported. Kappa-coefficients were calculated to evaluate consistency of the two parallel screening methods for the detection of low avidity HPV16/18 antibodies. The proportions of low-avidity HPV16 and HPV18 antibodies by the titration method were calculated, and the crude odds ratios (OR) with 95% confidence interval (CI) of low-avidity antibodies according to smoking status were estimated using logistic regression. All statistical analyses were performed with SPSS 16.0 and STATA 8 (Stata Corp., College, Texas, USA).

## Results

Demographic characteristics of the entire vaccinated cohort have been published previously [17]. In the present pilot study, we included 216 Finnish trial participants with unblinded vaccine codes, and who had complied with the full three-dose vaccination schedule. Out of 103 participants in the HPV16/18 vaccine group, 46.6% (48) were smokers compared to 56.6% (64) in the control group (n = 113).

Mean anti-HPV16 and HPV18 antibody levels at month 7 post-vaccination were very high in the HPV16/18 vaccine group as compared to the control (HAV vaccine) group or to baseline (Table 1). When the anti-HPV16 and anti-HPV18 antibody levels between non-smokers and smokers were compared, the mean absorbance of anti-HPV16 antibodies was 1.97 (±0.78) among non-smokers and 1.88 (±0.73) among smokers. Mean absorbance of anti-HPV18 antibodies was 1.44 (±0.85) among non-smokers and 1.36 (±0.76) among smokers. The observed differences between the smokers and non-smokers were not statistically significant (Table 1).

**Table 1:** Comparison of mean absorbance level and mean antibody titers with standard deviation (SD) of anti-HPV16 and HPV18 antibodies between non-smokers and smokers by vaccination group

	**HPV vaccine (n = 103)**			**HAV vaccine (n = 113)**		
	Non-smokers (n = 55)* mean (±SD)	Smokers (n = 48)* mean (±SD)	**p-value†**	Non-smokers (n = 49)* mean (±SD)	Smokers (n = 64)* mean (±SD)	**p-value†**
**HPV16**						
*Antibody absorbance*						
Baseline	0.12 (±0.08)	0.29 (±0.52)	0.2	0.21 (±0.43)	0.18 (±0.29 )	0.9
Month 7	1.97 (±0.78)	1.88 (±0.73)	0.4	0.01 (±0.04)	0.01 (±0.02)	0.7
**HPV18**						
*Antibody absorbance*						
Baseline	0.07 (±0.06)	0.18 (±0.008)	0.1	0.1 (±0.08)	0.10 (±0.14)	0.8
Month 7	1.44 (±0.85)	1.36 (±0.76)	0.6	0.01(±0.03)	0.01 (±0.03)	0.7

*Non-smokers (Cotinine <20 ng/ml) and smokers (cotinine > =20 ng/ml).

^†^p-value derived from Mann–Whitney test to evaluate mean absorbance difference between non-smokers and smokers.

We also evaluated in the HPV16/18 vaccine group the quality, i.e. the avidity of vaccine-induced HPV16 and HPV18 antibodies. In the screening phase, where 4 M urea or 1 M NH4CSN were used as chaotropic agents, reasonably high kappa-coefficients of consistency for presence of low avidity HPV antibodies (0.7 for low avidity HPV16 antibodies; 0.6 for low avidity HPV18 antibodies) were observed in 27 and 17 individuals, respectively.

By the reference approach (titration method using 4 M urea), 13 out of the 27 samples with screen-detected HPV16 samples were confirmed to have low avidity, whereas three of the 17 screen-detected HPV18 samples had low-avidity HPV18 antibodies (Table 2). Eleven of the 16 individuals with low-avidity HPV16/18 antibodies were smokers. They tended to have an increased risk of having low avidity HPV16/18 antibodies (OR 3.0, 95% confidence interval (1.0-9.3) (Table 2).

**Table 2:** Detection of persistent low avidity HPV16 or HPV18 antibodies at month 7 stratified by smoking status

	**Low avidity (= < 40%)**	**High avidity (>40%)**	**OR, 95% CI**
**HPV16**			
Non-smokers (n = 55)	4 (31%)	51 (57%)	1
Smokers (n = 48)	9 (69%)	39 (43%)	2.9 (0.8-10.3)
**HPV18**			
Non-smokers (n = 55)	1 (33%)	54 (54%)	1
Smokers (n = 48)	2 (67%)	46 (46%)	2.8 (0.2-31.6)
**HPV16/18**			
Non-smokers (n = 55)	5 (31%)	50 (57%)	1
Smokers (n = 48)	11 (69%)	37 (43%)	3.0 (1.0*-9.3)

*Exact value = 0.95, and 40% is the cut-off of low avidity.

## Discussion

Evaluating the influence of HPV co-factors, particularly tobacco smoking, in the induction of robust post-vaccination anti-HPV immune response is timely [2]. We found that vaccinated young women, who smoked had similar levels of anti-HPV16 and HPV18 antibodies compared to vaccinated, non-smoking women 7 months post-vaccination, whereas the avidity of HPV16/18 antibodies might be inferior in smokers.

In contrast to natural HPV infection, a prophylactic cervical cancer vaccine should induce long-term immune response with high and sustained local and systemic antibody levels [27]. The adjuvanted HPV16/18 induces significantly increased humoral response to the vaccine antigens [28]. In addition to 100% seroconversion rates, extremely high antibody levels following Cervarix™ vaccination have been evident since the interim analysis of the PATRICIA study, a large phase III trial of the AS04-adjuvanted bivalent HPV16/18 vaccine [16,17,29]. The geometric titers of vaccine induced antibodies against both HPV16 or HPV18 antigens are substantially higher and longer lasting than those in natural HPV16/18 infections at all time points post vaccination/infection [9,30].

Our pilot study in young women demonstrated no significant quantitative differences in vaccine-induced humoral immune response to HPV16/18 L1 VLPs among smokers compared to nonsmokers. The proportion of smoking women with apparently low avidity HPV16 antibodies was, however, approximately two times higher than in non-smoking women. Previous studies on HPV16/18 vaccine induced (maturation of) antibody avidity disagree on the existence of low-avidity antibodies [10,11,31]. In those studies confounding factors, e.g., differences in laboratory methods or smoking have not been considered. The applied chaotropic agent concentrations, re-optimized in our lab, had a minimal effect on the performance of the HPV16/18 VLP antigens in the standard ELISA assay (Namujju et al. unpublished).

Tobacco smoking, assessed via serum cotinine quantification, has been shown to have an adverse effect on immune response to natural HPV16/18 infections among young women followed-up for more than 5 years [12]. Although the underlying mechanism is unclear, researchers have demonstrated reduction in cervical density of Langerhans cells among smokers [32,33]. Tobacco smoking has also been associated with an increased probability of acquisition and a decreased probability of clearance of HPV infection [34,35] as well as decreased clearance of cervical lesions [13].

Cotinine is a highly sensitive and specific marker of current active or passive exposure to tobacco smoke [36-38]. The use of serum cotinine rather than questionnaire data integrates a number of aspects of the exposure including tobacco composition, uptake, distribution, and individual differences in metabolism [38]. Furthermore, it is the inhaled dose of tobacco smoke that is directly related to the development of tobacco-related disorders [26]. Thus, biochemical assessment of tobacco exposure underlines the validity of our observations.

Duration of HPV vaccine induced immune response and its sustained efficacy in preventing HPV infection and cervical neoplasia are under close surveillance [2]. Some studies have shown that vaccine induced antibody levels decline with time. Although this has been suggested also for the bivalent HPV16/18 vaccine we have not observed it in our current study due to the short duration. In a study by Olsson et al. [39] it was suggested that although the HPV18 antibodies declined over time, the vaccinees remained protected, pointing to the importance of factors other than antibody levels in long-term vaccine protection. Sustainable, high avidity IgG response could be a surrogate marker or indirect indicator of T-cell help post-vaccination, which may or may not be affected by smoking habit. Our study was limited by the small numbers of study participants.

## Conclusion

Our pilot results suggest that smoking may not interfere with the quantity, but affects the quality of HPV16/18 vaccine-induced peak IgG antibodies. Our findings warrant further large studies on the quality and duration of HPV16/18 vaccine induced antibody response.

## Competing interests

ML and JP have obtained grants from Merck & Co. Inc., and GlaxoSmithKline Biologicals through their employers. The other authors have no conflicts of interest to disclose.

## Authors’ contributions

PBN, ASK, HMS, ML designed the study; PBN, EP, LH conducted antibody avidity analysis; PBN, MM, ASK, ML participated in statistical analysis; PBN, EP, ML drafted manuscript, PBN, EP, ASK, HL, MM, HMS, RK, DA, JP, KH, ML contributed scientifically to the manuscript and approved the final manuscript.
